# Sunlike White Light-Emitting Diodes Based on Rare-Earth-Free Luminescent Materials

**DOI:** 10.3390/ma15051680

**Published:** 2022-02-23

**Authors:** Amador Menéndez-Velázquez, Dolores Morales, Ana Belén García-Delgado

**Affiliations:** Photoactive Materials Research Unit, IDONIAL Technology Center, 33417 Avilés, Spain; dolores.morales@idonial.com (D.M.); ana.delgado@idonial.com (A.B.G.-D.)

**Keywords:** solid-state lighting (SSL), white light-emitting diode (WLED), color rendering index (CRI), rare-earth-free luminescent materials, luminescent organic materials, spectral converters

## Abstract

Solid-state lighting (SSL) sources based on light-emitting diodes represent the new generation of highly efficient illumination systems that significantly impact energy-saving. The development of white light-emitting diodes (WLEDs) with a combination of high color rendering index (CRI) and high deep-red color rendering R_9_ is an important challenge in the field of solid-state lighting. On the other hand, most WLEDs use rare-earth inorganic luminescent materials. The annual demand for rare-earth metals has doubled to 125,000 tons in 15 years, and the demand is projected to reach 315,000 tons in 2030. The explosion in demand for these materials, combined with a monopolistic supply source, represents a real risk for the development of WLEDs in the next few years. Luminescent organic materials are a relevant and promising alternative. Here, we report a WLED with a very high CRI of 95.7 and R_9_ of 78.7, obtained using a combination of a blue LED chip (excitation source) and two organic luminescent dyes (Coumarin 6 and Lumogen Red) acting as spectral converters in a multilayer remote phosphor configuration. To the best of our knowledge, this is the first rare-earth-free WLED with such high values of CRI and R_9_.

## 1. Introduction

According to the US Department of Energy, lighting consumes 15% of the world’s total energy and is responsible for 5% of the world’s total greenhouse gas emissions. Population growth and urbanization are expected to increase lighting demand by 50% by 2030. One easy way to reduce this carbon footprint is to switch to energy-efficient light sources.

Incandescent and fluorescent lamps rely on heating a wire filament until it glows or send an electric discharge through an ionized gas, respectively, which usually accompanies large energy losses. Energy-efficient solid-state light sources, in particular light-emitting diodes (LEDs), offer an alternative approach for illumination with high efficiency to significantly reduce worldwide energy consumption [1,2,3,4]. LEDs rely on the emission of light through a spontaneous recombination process of the electron–hole pairs produced from current injection with small energy losses.

In 1961, the first inorganic semiconductor LED was fabricated at Texas Instruments. This device emitted near-infrared (NIR) radiation because the used semiconductor is InGaP with a bandgap of 1.37 eV. One year later, scientists at General Electric Company invented the first LED device that emitted visible red light instead of infrared light using GaAsP as a semiconductor [5]. Red, yellow, and green LEDs were commercialized in the early 1960s and 1970s using GaAsP [5] and GaPN [6]. Considering that As-based III–V compounds are used in red LEDs and P-based III–V compounds are used in green LEDs, it should be easy to predict which colors can be obtained from which element from the periodic table. At that time, many researchers believed that blue LEDs could be realized using N-based III–V compounds, i.e., GaN. However, growing bulk GaN crystals from a solution is a difficult task. Very high pressure and temperature are needed, comparable to those needed for the growth of diamonds or even higher-quality crystals [7,8].

From 1993 to 1996, Dr Nakamura, an employee of Nichia Chemical Industries, developed the world’s first high-brightness GaN-based blue LED [9]. Then, white light-emitting diodes (WLEDs) were developed by covering a blue LED with YAG:Ce as a phosphor-converted LED (pc-LED) to generate a pseudo-white light (i.e., mixing the original emitted and unconverted blue light and the converted yellow light). This development from Nakamura et al. enabled the use of LEDs in everyday applications for general lighting. For this breakthrough achievement, he received the 2014 Nobel Prize in Physics [10] with Isamu Akasaki and Hiroshi Amano.

WLEDs can also be fabricated by combining blue, green, and red LEDs [11]. However, due to the differences in the driving voltage, temperature, and aging characteristics of differently colored LEDs, developing WLEDs based on the three primary colors is not very practical. Existing commercial white light LED products mainly use blue or UV LEDs as the first-level light source and phosphors (light-conversion materials) to span the emission across the visible spectrum.

Currently, the most widely used phosphors are based on rare-earth elements (REEs) [12]. However, there are several drawbacks to the REE supply chain because rare-earth production that is concentrated in a few geographical regions accounts for over 95% of the world’s production of rare-earth elements [13]. For this reason, valid alternatives to REEs in photonic devices [14,15], with particular regard to lighting systems, are strongly required.

Organic luminescent materials [16] are promising candidates as spectral converters for photonic technologies with applications in different fields, such as photovoltaics [17,18], farming [19,20], ophthalmology [21], etc., due to their promising features such as solution processability, cost-effectiveness, and low toxicity. LED lighting can also benefit from the use of luminescent organic materials [22,23]. The right choice of UV/blue LED and luminescent molecular system (phosphors) is key to achieving enhanced emissions spanning the entire visible region along with good quality light.

In this communication, we present a WLED device based on a blue LED chip and two luminescent organic dyes. This device recreates the solar spectrum in terms of colorimetric parameters such as CRI and R_9_, reaching very high values for these parameters.

## 2. Materials and Methods

Most white LEDs on the market use blue LED chips made from GaN-based materials and some type of phosphor coating, placed either directly on the chips or separated from the chip (remote phosphor configuration) [24]. These WLED sources emit light with characteristic spectral distributions, which depend on the nature of the excitation source and the phosphors used. WLEDs provide a much higher degree of freedom to tune the light emission spectrum compared to conventional lighting technologies. This freedom provides new opportunities to optimize these WLED-based light sources. In this article, we report the development of 39 different WLEDs. They are based on a blue LED and a set of luminescent organic materials in remote phosphor configurations working as spectral converters.

### Selected Excitation Source and Materials

**Figure 1:**
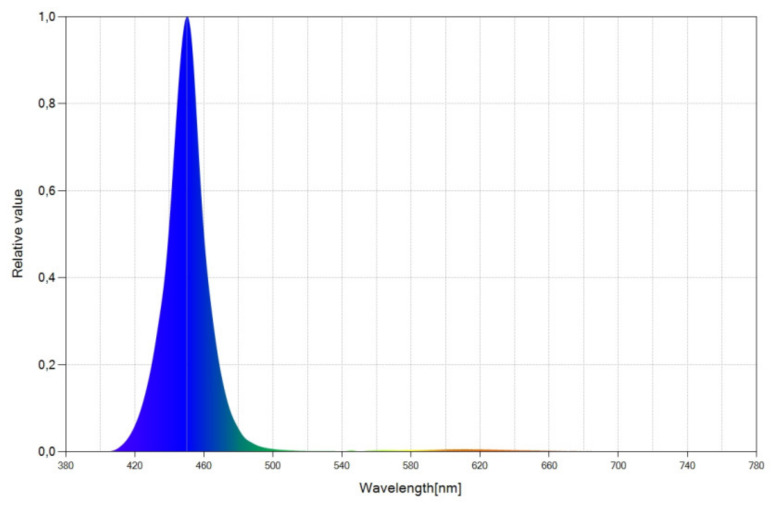
Spectral power distribution (SPD) chart of the selected blue LED.

As an excitation source, we used an LED chip whose relative spectral power distribution (SPD) chart is shown in Figure 1. SPD provides the emitted power as a function of the wavelength. The LED emits light in the (410–500 nm) range, which mostly corresponds to the blue range of the spectrum, with an emission peak at 450 nm.

Regarding the luminescent organic dyes, they are incorporated into a polymer matrix with high optical transparency, such as PMMA. These composite systems (polymeric matrix and luminescent species) are deposited on glass in the form of thin films. The thin films work as luminescent filters that spectrally convert light into longer wavelengths, therefore expanding the spectrum of the emitted light, with respect to the blue LED chip, to cover a larger fraction of the visible part of the spectrum. Each organic dye is incorporated into a different single layer. We developed monolayer and multilayer WLEDs with different dye concentrations to have greater versatility in the spectral conversion, thus achieving a very high-quality light.

**Figure 2:**
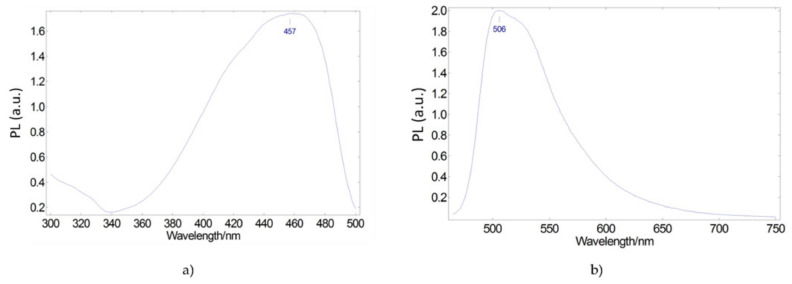
Photoluminescent excitation (**a**) and emission (**b**) spectra of Coumarin 6 green-emitting converter embedded in a PMMA matrix.

We begin by looking for a luminescent organic dye whose excitation spectral range overlaps with the emission spectrum of the selected blue LED. Coumarin 6 is a molecule that meets this requirement. Figure 2 shows its excitation and emission spectra. As shown in Figure 2a, the visible excitation spectrum extends from the ultraviolet to 500 nm, with an excitation peak at 457 nm, very close to the emission peak of the blue LED. The emission spectrum (Figure 2b) presents a peak at 506 nm, extending the emission range of the blue LED + phosphor system with respect to the pure blue LED.

**Figure 3:**
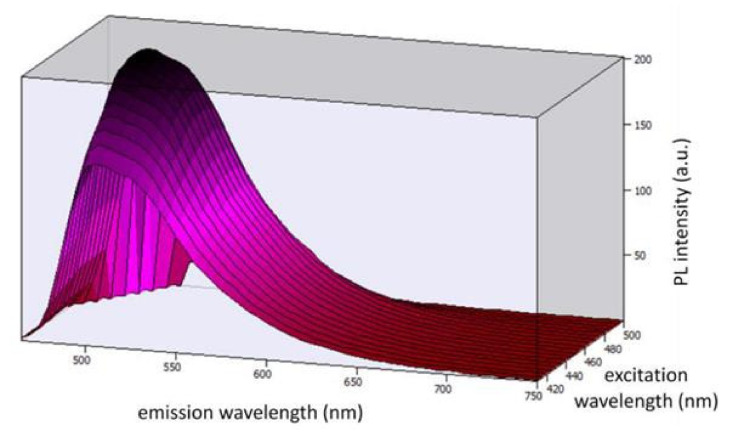
Three-dimensional photoluminescent spectra under the excitation wavelengths from 410 nm to 500 nm.

Figure 3 shows the 3D photoluminescent (PL) spectra under the excitation wavelengths from 410 nm to 500 nm (corresponding to the emission range of the blue LED chip). By observing the spectra, we can conclude that Coumarin 6 embedded in PMMA can be effectively excited by the selected blue LED chip.

**Figure 4:**
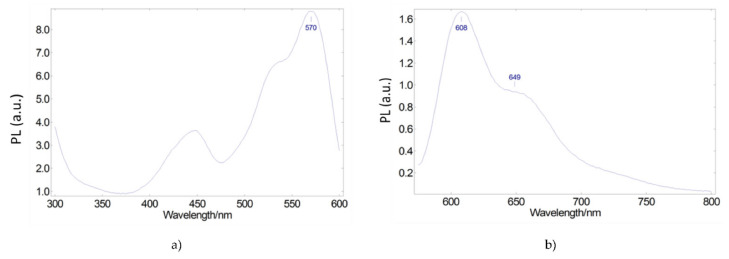
Photoluminescent excitation (**a**) and emission (**b**) spectra of Lumogen Red red-emitting converter embedded in a PMMA matrix.

We then look for a second luminescent organic dye that allows us to further extend the spectral range of emitted light. Lumogen Red is a molecule with a broad excitation spectrum, which can partially absorb both the light emitted by the blue LED chip and the light corresponding to the Coumarin 6 emission spectrum. Figure 4 shows its excitation and emission spectra. As shown in Figure 4a, the visible excitation spectrum extends from ultraviolet to 600 nm, with an excitation peak at 450 nm, overlapping with the emission peak of the blue LED, and another excitation peak at 570 nm, overlapping with the Coumarin 6 emission spectrum. The emission spectrum (Figure 4b) presents two peaks, at 608 nm and 649 nm, allowing the spectral range of the emitted light to be further extended.

**Figure 5:**
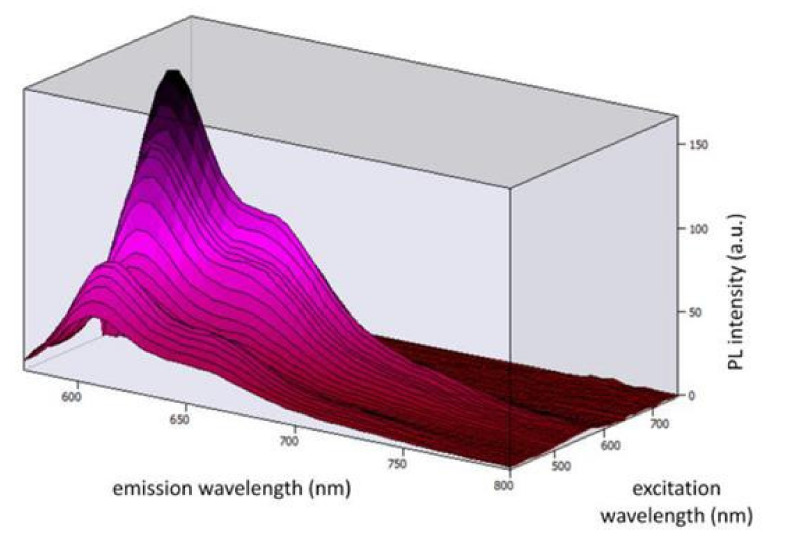
Three-dimensional photoluminescent spectra under the excitation wavelengths from 410 nm to 740 nm.

Figure 5 shows the 3D PL spectra under the excitation wavelengths from 410 nm to 740 nm (corresponding to the emission of the blue LED chip and the Coumarin 6 dye). By observing the spectra, we can conclude that Lumogen Red embedded in PMMA can be excited by the blue LED + Coumarin 6 system.

Another key property of a luminescent material acting as spectral converter is the quantum yield (QY), which is defined as the ratio of emitted photons to absorbed photons. These luminescent organic dyes (Coumarin 6 and Lumogen Red) possess high quantum yields both in solution and solid state. QY of Coumarin 6 and Lumogen Red embedded in PMMA is 76% and 97%, respectively.

## 3. Results and Discussion

By combining the luminescent species Coumarin 6 (C6) and Lumogen Red (LRed) in the same layer or different layers and varying their concentrations, it is possible to achieve different spectral conversions. The different concentrations are expressed as weight percent with respect to the host material (PMMA).

We developed 39 different WLEDs, starting from the same blue LED previously described (see Figure 1), and 39 different configurations of spectral converters. We will group the different spectral converters into three categories: monolayer molecular systems with just one luminescent dye (Section 3.2), multilayer (two layers) molecular systems with two different luminescent dyes, one dye in each layer (Section 3.3), and inverted multilayer luminescent molecular systems (Section 3.4). These inverted systems (Section 3.4) are the same molecular systems as those of Section 3.3, but the position of the layers is inverted with respect to the blue LED chip.

By measuring the optical properties of the WLEDs, it is possible to compare the different developed WLEDs and optimize the configuration to achieve a WLED with high light quality. In Section 3.1, we describe and justify the optical characterizations that will be carried out in the following sections with the different WLEDs developed.

### 3.1. Optical Characterization

SPD, CRI, and R_9_ parameters will be taken into account during the WLED optimization process. CCT will be considered only at the end to ensure that the optimized WLEDs are within CCT acceptable limits and in accordance with values on the market.

#### 3.1.1. Spectral Distribution Power (SPD)

The spectral power distribution (SPD) chart visually represents the light spectrum emitted by a light source; it gives the emitted power as a function of the wavelength. SPD charts are a practical way to compare the quality and type of light created by different light sources. The chart shows which wavelengths of light (measured in nanometers) the illuminated regions receive. The spectral power distribution is considered the “fingerprint” of an LED system, from which other parameters, such as CRI, R_9_ and CCT, can be determined.

#### 3.1.2. Color Rendering Index (CRI)

The color rendering index (CRI)—defined by the Commission Internationale de l’Éclairage (CIE, International Commission on Illumination)—is a quantitative measure of a light source’s ability to accurately reproduce the color of an illuminated object, compared to a reference light source, such as the sunlight. The standard CRI points, namely R_1_–R_8_, are derived from CIE 1974 test color samples (TCS), and arithmetical means of these values (R_a_) are usually used to report the ability to reproduce the color accurately. The terms CRI and R_a_ are used interchangeably in the lighting industry. All eight TCSs used in the computation of R_a_ are pastels with relatively low color saturation. R_a_ alone is therefore not a good indicator for the quality of light in many applications where more saturated color objects are involved.

#### 3.1.3. Red Color Rendering Index (R_9_) and Other CRI Points

A special red color rendering index (R_9_) has been added to LED lighting specifications to supplement R_a_. The Energy Star program (V2.0) in the United States requests that all LED lamps have an R_9_ larger than zero. Deep-red color is prevalent in hues such as lighted meat, fish, vegetables, and fruit in supermarkets, clothes in display windows, human skin, and surgical procedures. The special CRI points (R_9_–R_15_) are usually represented in the CRI points diagrams.

#### 3.1.4. Correlated Color Temperature (CCT)

Correlated color temperature (CCT) in kelvins (K) indicates the color of a source of light by comparing it with a black body color, which is a “theoretically perfect radiant” (an object whose light emission is only due to its temperature). As with any other incandescent body, the black body changes its color as its temperature increases, acquiring a red matte tone at the beginning, which later changes to light red, orange, yellow, and finally white, bluish-white, and blue. For example, the color of a candle flame is similar to a black body heated at about 1800 K. Then, the flame is said to have a “color temperature” of 1800 K.

### 3.2. Monolayer Luminescent Molecular Systems

Using the selected blue LED (see Figure 1), we developed 3 WLEDs with the organic luminescent dye Coumarin 6 integrated into a single layer with different concentrations for each WLED. Table 1 summarizes the characteristics of the developed WLEDs.

As shown in the table, using Coumarin 6 dye with a concentration ≥2.3%, a white light with a CRI above 72 is achieved. The best device is WLED-2 with a CRI of 74.8; however, in this WLED and other WLEDs R_9_ take negative values.

**Figure 6:**
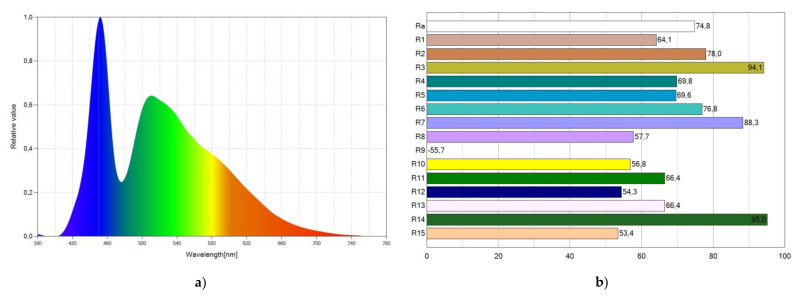
Spectral power distribution (SPD) chart (**a**) and CRI points (**b**) of WLED-2.

Figure 6a shows the spectral distribution power (SPD) of WLED-2. This figure also shows two peaks in the spectrum from the blue LED and the Coumarin 6 luminescence. Figure 6b shows the corresponding CRI points.

### 3.3. Multilayer Luminescent Molecular Systems

We used the same blue LED (see Figure 1) and developed 18 WLEDs with two luminescent species, Coumarin 6 and Lumogen Red. Each organic dye is in a different layer. The layer that contains Coumarin 6 is called Layer 1, and the layer that contains Lumogen Red is called Layer 2. In the different configurations developed in this section, Layer 1 (Coumarin 6) is the closest to the blue LED chip, and Layer 2 (Lumogen Red) is the furthest away. Using two dyes (Coumarin 6 and Lumogen Red), instead of just one (Coumarin 6), we pursued a double objective: on the one hand, to expand the emission spectrum to improve the CRI; on the other hand, to increase the red component of the spectrum to achieve a high value of R_9._

Table 2 shows the different formulations of the developed spectral converters and the CRI and R_9_ values of the resulting WLEDs. As shown in the table, there are several WLEDs with high values of CRI and high positive values of R_9_. Regarding the CRI, the best value (96.3) was achieved with WLED-11, providing a positive and good value of R_9_ (66.3) at the same time. If we consider the R_9_ parameter, the maximum value (81.2) was reached in the WLED-18, but the CRI value (88) was reduced by 8.3 units with respect to WLED-11.

Therefore, unless we need a very specific application where deep reds are very important, we can conclude that WLED-11 provides better light quality than WLED-18. However, both provide excellent CRI and R_9_ values.

**Figure 7:**
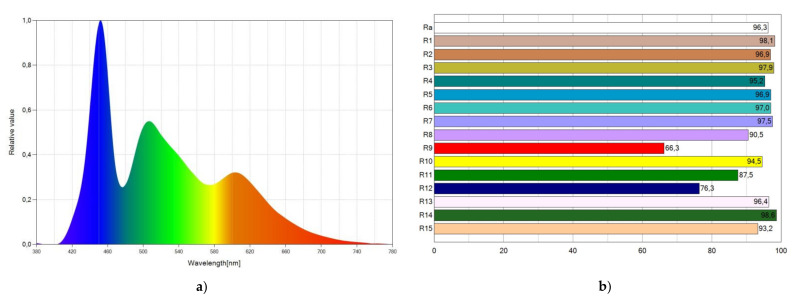
Spectral power distribution (SPD) chart (**a**) and CRI points (**b**) of WLED-11.

Figure 7 shows the SPD chart of WLED-11 and the CRI points. As shown in Figure 7a, the spectrum of emitted light expands to the entire visible spectrum, which explains the high CRI value achieved. SPD shows three peaks due to the light emission from the blue LED chip, from Coumarin 6, and from Lumogen Red. Figure 7b shows the CRI points. In general, most of them reach very high values, with the value of R_9_ being the lowest.

### 3.4. Inverted Multilayer Luminescent Molecular Systems

In this section, we describe the manufacture of another 18 WLEDs. These WLEDs result from inverting the layers in the WLEDs described in the previous section. Now Layer 2 (which contains Lumogen Red) is the closest to the blue LED chip, and Layer 1 (which contains Coumarin 6) is the furthest from the blue LED chip. Table 3 shows the different formulations of the developed spectral converters and the CRI and R_9_ values of the resulting WLEDs. The best CRI value (95.7) was achieved with the WLED-11-I, also reaching an excellent value of R_9_ (78.7). The best value of R_9_ (87.1) was achieved with WLED-18-I, but in this case, the value of CRI (85.4) was notably sacrificed. Therefore, we can conclude that the best WLED is WLED-11-I, since it achieves very high values for both parameters (CRI and R_9_).

**Figure 8:**
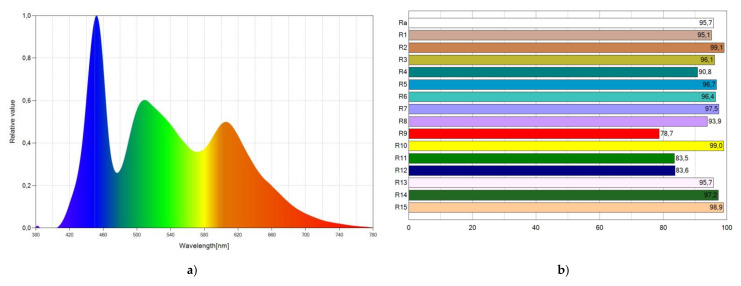
Spectral power distribution (SPD) chart (**a**) and CRI points (**b**) of WLED-11-I.

Figure 8 shows the SPD chart of WLED-11-I, as well as the CRI points. As shown in Figure 8a, the spectrum of emitted light is expanded to the entire visible spectrum, which explains the high CRI value achieved. SPD shows three peaks corresponding to the light emission from the blue LED chip, from Coumarin 6, and from Lumogen Red. Figure 8b shows the CRI points. In general, most of them reach very high values, including the critical R_9_ parameter, which reaches the value of 78.7.

### 3.5. High-Quality WLEDs

In this section, we compare the best WLEDs obtained in the previous sections: WLED-11 and WLED-18 (multilayer molecular systems), and WLED-11-I and WLED-18-I (inverted multilayer molecular systems). Table 4 shows these WLEDs and their optical properties.

If we consider the CRI and R_9_ values simultaneously, WLED-11-I is the one that provides the best values. A CRI of 95.7 and an R_9_ of 78.7 are considered excellent values in the LED industry. In particular, R_9_ is far more difficult to achieve a high score compared to the other R_i_ values that comprise the CRI calculations. Therefore, an R_9_ score of 50 or above is usually considered “good”, while an R_9_ score of 75 or above would be considered “excellent”.

**Figure 9:**
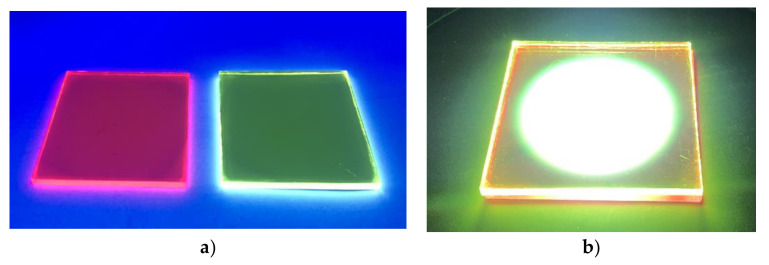
(**a**) Photoluminescent red-emitting and green-emitting converters embedded in a PMMA matrix and coated on glass under exposure to blue light. (**b**) White light emitted by the WLED-11-I device.

On the other hand, the correlated color temperature of WLED-11-I (7133 K) is within the market standards. All this evidence validates the very high light quality of this WLED, which provides the best values reported so far in the scientific literature using organic materials as spectral converters. Figure 9 shows photographs of the WLED device and the white light emitted.

WLEDs with both high CRI and R_9_ values, as achieved in this article, are required and attracting much more attention presently. This has also been possible by making use of organic luminescent materials, free of rare-earth elements and not subject to problems of demand, high cost, monopolies, or geopolitical tensions, which still suggests another great added value. Preliminary accelerated aging studies show a good photostability of these dyes (see Supplementary Materials).

## 4. Conclusions

White light-emitting diodes (WLEDs) are considered one of the most promising next-generation lighting technologies because they significantly reduce global power requirements. WLEDs have high luminous efficiency and low power consumption. Current WLED technologies typically use a single semiconductor chip to produce light, usually blue, and then rely on rare-earth luminescent materials to shift the color to white. Rare-earth materials are subject to a monopoly, and their distribution and high cost could put the LED industry in serious danger.

Organic luminescent materials are gaining prominence in the photonics industry. Among their many applications, their possible use in artificial lighting as spectral converters is gaining relevance, and they are a promising alternative to rare-earth elements. By employing organic materials in WLEDs, it is possible to accomplish a successful color rendition of the illuminated objects.

In this article, we have reported two organic dyes, acting as remote phosphors and pumped by a blue LED chip. The resulting WLED emits a white light with simultaneously very high values of CRI (95.7) and R_9_ (78.7). To the best of our knowledge, these are the best simultaneously reported values for a WLED using organic materials as spectral converters. This research shows the enormous potential of this kind of material in the LED industry of the future.

## Figures and Tables

**Table 1:** Description of the WLEDs (1, 2, and 3) and optical properties.

WLED	% Dye		CRI	R_9_
WLED-1	C6	2.3%	72.1	−69.8
WLED-2	C6	2.5%	74.8	−55.7
WLED-3	C6	3%	73.9	−58.1

**Table 2:** Description of the WLEDs (4 to 21) and optical properties.

WLED	% Dye (Layer 1)		% Dye (Layer 2)		CRI	R_9_
WLED-4	C6	2.3%	LRed	0.5%	83.2	−9.7
WLED-5	C6	2.3%	LRed	1%	92.5	43.1
WLED-6	C6	2.3%	LRed	2%	87.7	67
WLED-7	C6	2.3%	LRed	3%	71.6	−6.4
WLED-8	C6	2.3%	LRed	4%	64.2	−19.9
WLED-9	C6	2.3%	LRed	5%	35	−100
WLED-10	C6	2.5%	LRed	0.5%	86.4	−7.9
WLED-11	C6	2.5%	LRed	1%	96.3	66.3
WLED-12	C6	2.5%	LRed	2%	85.8	56.4
WLED-13	C6	2.5%	LRed	3%	69.7	−12.1
WLED-14	C6	2.5%	LRed	4%	56.8	−70.5
WLED-15	C6	2.5%	LRed	5%	31.6	−100
WLED-16	C6	3%	LRed	0.5%	83.9	−6.7
WLED-17	C6	3%	LRed	1%	92.4	36.6
WLED-18	C6	3%	LRed	2%	88	81.2
WLED-19	C6	3%	LRed	3%	73.5	31.1
WLED-20	C6	3%	LRed	4%	66.4	9.0
WLED-21	C6	3%	LRed	5%	44	−42.4

**Table 3:** Description of the WLEDs (4-I to 21-I) and optical properties.

WLED	% Dye (Layer 1)		% Dye (Layer 2)		CRI	R_9_
WLED-4-I	LRed	0.5%	C6	2.3%	85.1	−0.5
WLED-5-I	LRed	1%	C6	2.3%	95.3	56.0
WLED-6-I	LRed	2%	C6	2.3%	85.7	75.4
WLED-7-I	LRed	3%	C6	2.3%	75.5	36.2
WLED-8-I	LRed	4%	C6	2.3%	71.0	22.7
WLED-9-I	LRed	5%	C6	2.3%	63.3	8.5
WLED-10-I	LRed	0.5%	C6	2.5%	88.7	22.2
WLED-11-I	LRed	1%	C6	2.5%	95.7	78.7
WLED-12-I	LRed	2%	C6	2.5%	80.9	51.7
WLED-13-I	LRed	3%	C6	2.5%	70.5	15.1
WLED-14-I	LRed	4%	C6	2.5%	68.3	11.0
WLED-15-I	LRed	5%	C6	2.5%	59.7	−1.7
WLED-16-I	LRed	0.5%	C6	3%	86.4	4.6
WLED-17-I	LRed	1%	C6	3%	95.7	57.7
WLED-18-I	LRed	2%	C6	3%	85.4	87.1
WLED-19-I	LRed	3%	C6	3%	78.3	75.1
WLED-20-I	LRed	4%	C6	3%	73.5	67.0
WLED-21-I	LRed	5%	C6	3%	66.2	54.6

**Table 4:** Selection of the best WLEDs and optical properties.

WLED	% Dye (Layer 1)		% Dye (Layer 2)		CRI	R_9_	CCT (K)
WLED-11	C6	2.5%	LRed	1%	96.3	66.3	11,075
WLED-18	C6	3%	LRed	2%	88	81.2	6073
WLED-11-I	LRed	1%	C6	2.5%	95.7	78.7	7133
WLED-18-I	LRed	2%	C6	3%	85.4	87.1	4036

## Data Availability

The data supporting the findings of this study are available within the article and the Supplementary Materials.

## Supplementary Materials

The following supporting information can be downloaded at: https://www.mdpi.com/article/10.3390/ma15051680/s1. Figure S1. Absorption (a) and pholuminiscence (b) spectra of Coumarin 6 samples before and after UV exposure; Figure S2. Absorption (a) and pholuminiscence (b) spectra of Lumogen Red samples before and after UV exposure.
