# Supplementary material for: Sunlike White Light-Emitting Diodes Based on Rare-Earth-Free Luminescent Materials
Source: Materials (Basel). 2022 Feb 23;15(5):1680. doi: 10.3390/ma15051680 (PMC8911033; doi:10.3390/ma15051680)
Supplement: Supplementary file 1 [file materials-15-01680-s001.zip › materials-1560295-supplementary.pdf]

materials

Supplementary Materials

Sunlike White Light-Emitting Diodes Based on Rare-Earth-Free Luminescent Materials

Amador Menéndez-Velázquez *, Dolores Morales and Ana Belén García-Delgado

Photoactive Materials Research Unit, IDONIAL Technology Center, 33417 Avilés, Spain; dolores.morales@idonial.com (D.M.); ana.delgado@idonial.com (A.B.G.-D.)
* Correspondence: amador.menendez@idonial.com

Citation: Menéndez-Velázquez, A.; Morales, D.; García-Delgado, A.B. Sunlike White Light-Emitting Diodes Based on Rare-Earth-Free Luminescent Materials. *Materials* **2022**, *15*, 1680. https://doi.org/10.3390/ma15051680

Academic Editor: Hélène Serier-Brault

Received: 31 December 2021
Accepted: 21 February 2022
Published: 23 February 2022

Publisher's Note: MDPI stays neutral with regard to jurisdictional claims in published maps and institutional affiliations.

Copyright: © 2022 by the authors. Licensee MDPI, Basel, Switzerland. This article is an open access article distributed under the terms and conditions of the Creative Commons Attribution (CC BY) license (https://creativecommons.org/licenses/by/4.0/).

Preliminary Accelerated Aging Testing on the Luminescent Thin Films

Luminescent thin films coated on glass were subjected to accelerated UV radiation to check their photo-stability. The selected films for aging studies are:

- Thin film composed of Coumarin 6 green-emitting converter embedded in a PMMA matrix and coated on glass
- Thin film composed of Lumogen Red red-emitting converter embedded in a PMMA matrix and coated on glass

UV Aging Procedure

The samples were exposed to ultraviolet (UV) radiation with a wavelength peak of 340 nm during 8 hours at 45 °C. The irradiation intensity is 0.76 W/m^2.

Characterization Procedure

Absorption and photoluminiscence of the original and aged samples were evaluated.

Results and Discussion

a) Coumarin 6 green-emitting converter embedded in a PMMA matrix and coated on glass

Figure 1 shows the absorption and photoluminiscent spectra (before and adfter UV exposure) of a thin film composed of Coumarin 6 green-emitting converter embedded in a PMMA matrix and coated on glass. As show in Figure 1a, after 8h of UV exposure the absorption decrease a little bit. However, after UV exposure, the photoluminiscence (see Figure 1b), increases a little bit. Maybe there is some kind of minimal degradation and the intermediate species shows a higher fluorescence, but more studies need to be done before reaching these conclusions.

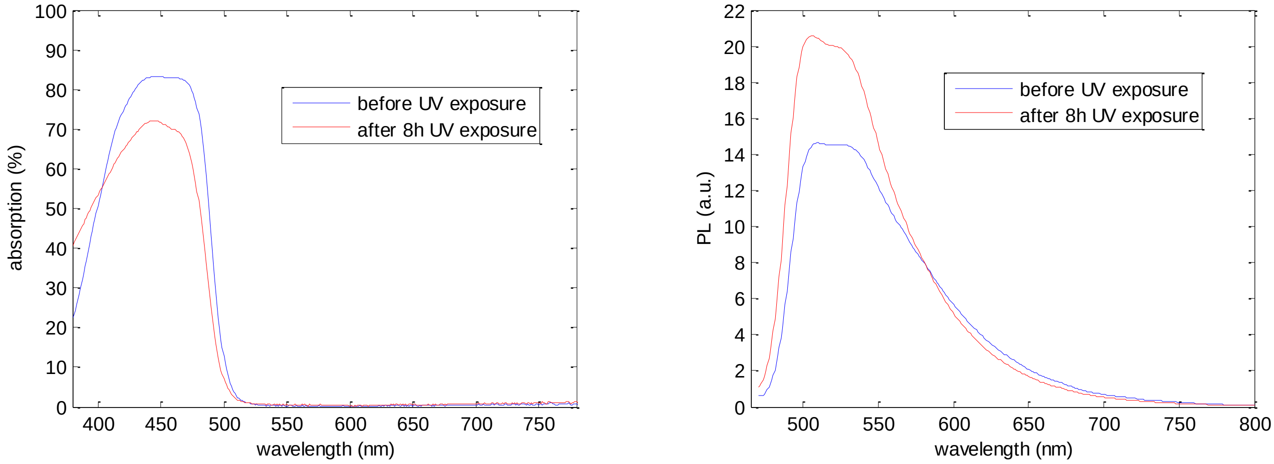

a) b)

Figure S1. Absorption (**a**) and pholuminiscence (**b**) spectra of Coumarin 6 samples before and after UV exposure.

b) Lumogen Red red-emitting converter embedded in a PMMA matrix and coated on glass

Figure S2 shows the absorption and photoluminiscent spectra (before and after UV exposure) of a thin film composed of Lumogen Red red-emitting converter embedded in a PMMA matrix and coated on glass. As show in absorption and photoluminescence spectra, there is not almost any change (before and after UV exposure) in the absorption and photoluminescence properties. Therefore we can conclude that there was no degradation of the Lumogen Red dye when exposed to UV radiation.

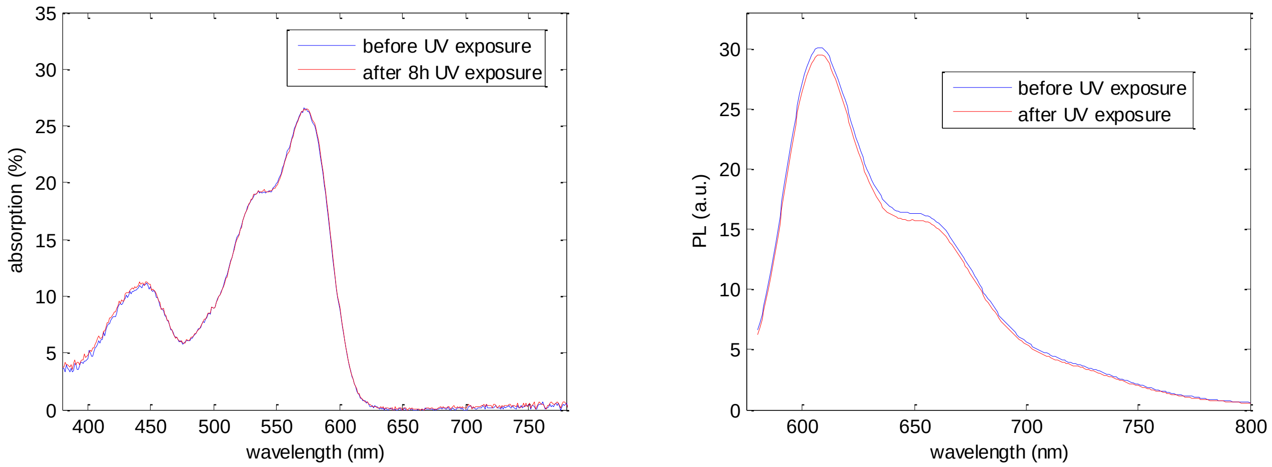

a) b)

Figure S2. Absorption (**a**) and pholuminiscence (**b**) spectra of Lumogen Red samples before and after UV exposure.

Conclusions

From this preliminary aging study we can conclude that Lumogen Red exhibits a very good photostability and Coumarin 6 shows a pretty good photo-stability in the manufactured luminescent films. However, further studies are required to check the photo-stability of these organic dyes and reach stronger conclusions.
